# Current state of the literature on mental health in Liberia: A systematic review

**DOI:** 10.4102/sajpsychiatry.v26i0.1502

**Published:** 2020-10-26

**Authors:** Kimberly Hook, Kanako Ando, Senait Ghebrehiwet, Benjamin Harris, Babawale Ojediran, Haniya Syeda, David Henderson, Christina Borba

**Affiliations:** 1 Department of Psychiatry, Boston University School of Medicine, Boston, MA, United States of America; 2 Department of Psychiatry, Boston Medical Center, Boston, MA, United States of America; 3 Department of Psychiatry, Massachusetts General Hospital, Boston, MA,United States of America; 4 A.M. Dogliotti College of Medicine, University of Liberia, Monrovia, Liberia

**Keywords:** Ebola, mental health disorders, Liberia, intervention and assessment, psychiatric interventions, substance use practices, adolescent

## Abstract

**Background:**

The Republic of Liberia recently experienced several events that resulted in wide-ranging societal impacts, including long-term civil war and an outbreak of Ebola. These types of events are linked to higher prevalence of mental disorders and psychosocial distress. As a result, it is likely that there is an increased prevalence of mental health disorders in the population.

**Aim:**

To assess and review the recent mental health literature in order to provide insight into existing mental health needs and effective or recommended interventions in post-conflict Liberia.

**Setting:**

Articles included in this study enrolled Liberians living in Liberia.

**Methods:**

A search of four databases was conducted for studies of any type that assessed mental health in Liberia between 01 January 2003 and 27 March 2019. After reviewing 363 articles, 21 articles were included in the final analysis. Articles were coded to identify common themes and needs.

**Results:**

The majority of studies used qualitative designs and were conducted in Monrovia, the capital city of Liberia. Common topics included adolescent mental health, intervention and assessment and post-conflict impacts. One article focused on mental health impacts after recovery from Ebola.

**Conclusion:**

Overall, there is a dearth of mental health literature that focuses on Liberia. This suggests ample opportunity for researchers to investigate mental health needs amongst the Liberian population and effective psychiatric interventions. Existing recommendations often focus on addressing adolescent health needs, including substance use practices. Opportunities for future research particularly related to needs of adult populations and to mental health impacts of Ebola, abound.

## Introduction

The Republic of Liberia, located on the coast of West Africa, recently endured several significant events that resulted in wide-ranging societal impacts. A longstanding 14-year civil war resulted in the loss of 250 000 lives and displaced 1.3 million people^1^, as well as destroyed the country’s health system and political stability^2^. Liberians experienced significant trauma and loss through ethnic killings, the forced servitude of child combatants and sexual violence.^3^ Approximately 340 000 children were orphaned as a direct result of the civil war^4^. Approximately 10 years after the Liberian civil war, the subsequent Ebola epidemic resulted in over 11 000 deaths across West Africa^5^, culminating in extreme suffering, emotional distress and economic collapse.^6^

Disasters, such as civil wars and public health events, often divert resources away from mental healthcare to address immediate needs and disaster relief. As war and life-threatening events are linked to higher prevalence of mental disorders and psychosocial distress^2,6,7^, it is likely that there is an increased prevalence of mental health disorders in the Liberian population stemming from these conflicts. Studies reveal untreated mental health problems amongst Liberians, which affect both well-being and quality of life.^3^ Much of Liberia’s health infrastructure, including mental health services, was severely impacted in past conflict, and significant challenges to meet these mental health needs remain to this day^8^.

In order to rebuild infrastructure and improve accessibility of mental health resources, it is necessary to take stock of the current landscape of mental health training, research and other initiatives. To our knowledge, no reviews have been conducted on previous mental health research in Liberia. In this study, we examine the existing evidence base and present findings on the current state of the literature regarding mental health and substance use in post-conflict Liberia, discuss gaps in knowledge and treatment and suggest future directions for research.

## Methods

With the assistance of an experienced health services librarian, a systematic review of the current literature contained within PubMed, PsycINFO, Embase and Web of Science (last updated on March 27, 2019) was conducted. Search terms included the following phrases: ‘mental health and Liberia’ and ‘substance abuse and Liberia’. Additionally, associated MeSH terms were included to account for variances in language; categories included substance-related disorders (all subheadings), mental health (all subheadings) and Liberia (all subheadings). This search returned 361 titles; two additional titles known to the authors were also included. We followed PRISMA guidelines^9^ to guide the search, analysis and reporting of data. We did not formally register this project on a public database of systematic reviews.

Reference lists of included articles were also double-screened by the authors; 28 additional abstracts were assessed as a result of this process, though no further publications were added for inclusion in the analysis. After duplicates were removed, 247 publications were screened at the level of title and abstract. Articles were independently screened and assessed for inclusion in this study by authors (KH and KA); any disagreements were resolved through discussion and consensus.

**FIGURE 1:**
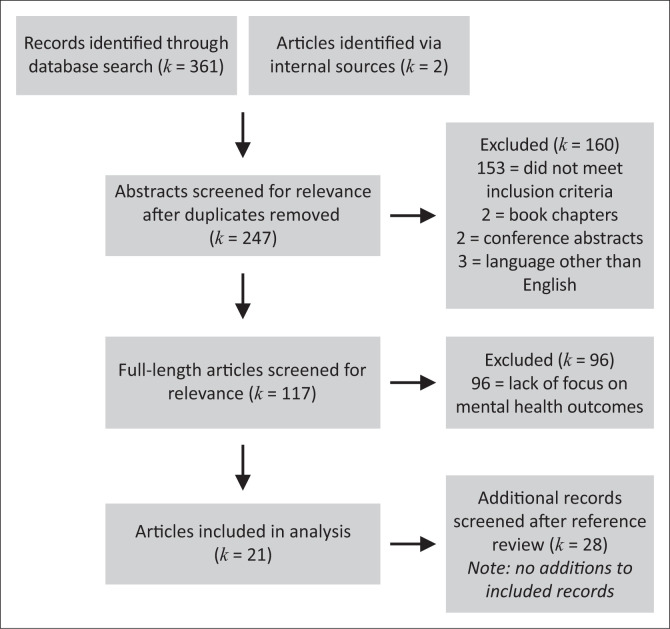
PRISMA diagram for the identification of articles.

Full-length articles were pulled for review if inclusion criteria were met. Inclusion criteria were as follows: (1) full-length article written in English; (2) published in a peer-reviewed journal or published dissertation; (3) published between 01 January 2003 and 27 March 2019 (i.e. to capture the literature in the immediate post-conflict to present range); (4) primary outcomes of the article focused on substance use and/or mental health, knowledge that impacted substance use or mental healthcare and/or mental health of the Liberian population, which excluded studies with a focus on policy or only indirect reference to mental health; (5) enrolled participants living in Liberia (i.e. not Liberian refugees in another country, in order to not be confounded by effects of migration on mental health) and (6) specific to the Liberian population (e.g. not foreign aid workers). Studies that grouped many countries together without differentiation (e.g. used the phrase ‘sub-Saharan Africa’ without further delineation between groups) or had a sole focus on child soldiers were excluded. In sum, 21 articles were included in this review. Figure 1 provides the synthesis of the review process, and Table 1 describes summary characteristics of included publications.

**TABLE 1:** Overview of included articles.

Authors (publication date)	Study site	Study design	Sample characteristics	Topics	Findings
Lekskes et al.^21^	Not specified	Qualitative (semi-structured interviews) Quantitative (survey)	*N* = 145 *Qualitative not specified* Sample: all female	Psychosocial interventions Females Sexual violence	Compared effectiveness of two psychosocial interventions amongst women who experienced war-related and sexual violence Trauma counseling most effective in treating PTSD compared to support and skills training
Johnson et al.^7^	Not specified; used systematic random sampling and cluster sampling	Qualitative (focus group testing) Quantitative (survey)	*N* = 1666 *Qualitative not specified* Sample: adults 18+ years old	Civil war PTSD Sexual violence Population characteristics related to mental health	Assessed impact of psychosocial trauma related to the Liberian civil war Adult combatants showed higher rates of PTSD, major depressive disorder and suicidal ideation compared to noncombatants Those who experienced sexual violence showed higher rates vs. those who did not
Galea et al.^25^	Nimba County (rural)	Qualitative (focus group testing) Quantitative (survey)	*N* = 1376 *Qualitative not specified* Sample: adults 19+ years old	PTSD	Examined the geographical distribution of PTSD Prevalence of PTSD was consistent with the geographic patterns of conflict across the country
Rockers et al.^26^	Nimba country (rural)	Quantitative (surveys)	*N* = 1434 Sample: adults 18+ years old	PTSD Role of communities	Examined association between community characteristics and post-traumatic stress symptoms in a resettled post-conflict population A higher mean number of traumatic experiences or displacement within the village was associated with a higher symptom score
Dominguez et al.^17^	Not specified	Qualitative (key informants)	*N* = 171 Sample: adults	Population characteristics related to mental health	Studied mental health after the civil war Young Liberians participate in high-risk behaviours (e.g. violence, substance abuse) Negative influence of peers and lack of respect for authority prevent youth from seeking treatment or building healthy relationships Protective factors, such as safety and support from family members, are limited
Harris et al.^11^	Monrovia (urban) Secondary schools	Quantitative (cross-sectional)	*N* = 802 Age (mean) = 19 years old (*SD*: 3) Sample: adolescents and adults (ages 12–36 years old)	Substance use Adolescents	Determined degree of substance use Most commonly reported substance was alcohol Marijuana more likely to be used amongst older respondents and men
Johnson et al.^27^	Not specified	Quantitative (surveys)	*N* = 549 Sample: adults; all former combatants	Civil war Population characteristics related to mental health	Examined association between previous head injury and mental health symptoms amongst former combatants Those with reported head injuries are more likely to show signs of major depressive disorder, suicidal ideation and substance abuse
Levey et al.^20^	Not specified	Qualitative (key informants)	*N* = 171 Sample: adults	Adolescents’ mental health needs	Assessed impact of traumatic events and treatment for youth Counselling, education and skills training were preferred by all age and gender groups Access to medical care, mental healthcare and education were described as necessary
Vinck and Pham^24^	Not specified (across 15 counties)	Quantitative (surveys)	*N* = 4501 Sample: adults	Trauma IPV Women Civil war impacts	Studied association of IPV and potentially traumatic war-related events Results showed association between IPV and symptoms of PTSD and depression for women Also revealed association between perpetrating IPV and symptoms of PTSD and depression for men
Ghailian^19^	Lofa County(rural)	Quantitative (surveys)	*N* = 193 Sample: children and adolescents (ages 10–20 years old)	Trauma Mental health of children	Explored association between adversity and prosociality in children who had experienced trauma Total trauma exposure was negatively associated with prosocial behaviour
Prust et al.^14^	Monrovia (urban)	Qualitative	*N* = 41 Sample: adults (ages 18–35 years old)	Substance use Adolescents	Outlined substance use and risk factors amongst high-risk groups Risk factors included participation in war, forced drug use, peer influence and economic and individual factors Consequences of substance use included individual consequences (dependence, physical health and social consequences) and community consequences (crime, violence, sexual risk)
Quiterio et al.^15^	Monrovia (urban) Secondary schools	Quantitative (cross-sectional)	*N* = 802 Sample: adolescents and adults (ages 12–36 years old)	Substance use Risky sexual behaviours Adolescents	Assessed substance use and sexual practices of youth Found association between alcohol use and engaging in sex and an increase in the number of sexual partners
Borba et al.^3^	Not specified Key informants	Quantitative (surveys)	*N* = 171 Sample: adults	Population characteristics related to mental health Adolescents	Examined mental health needs of Liberian youth Negative influences on youth mental health included war exposure, post-conflict sexual violence, poverty, infectious disease and parental death Protective factors, such as education, employment and positive social relationships, were hindered by the functional impairment
Levey et al.^4^	Monrovia (urban)	Qualitative	*N* = 75 Sample: adolescents (ages 13–18 years old)	Resilience Adolescents	Identified factors impacting resilience amongst youth Youth enrolled in school showed greater adaptive functioning, whilst participants who showed resilient outcomes displayed emotion regulation, cognitive flexibility, agency and social intelligence
Pullen et al.^16^	Monrovia (urban) Public schools	Qualitative (focus groups)	*N* = 72 Sample: adolescents	Substance use Adolescents	Studied behaviours, consequences and protective factors associated with substance use amongst youth Substance use was common as a means of coping School was a protective factor against substance use
Rabelo et al.^6^	Monrovia (urban)	Qualitative (focus groups)	*N* = 17 Sample: discharged patients from Ebola Treatment Unit	Population characteristics related to mental health Impacts of Ebola	Conducted an evaluation of mental health outcomes for survivors of Ebola Reported risk factors (e.g. stigma, exposure to death, isolation from family, negative interactions with staff, etc.) and protective factors (e.g. positive staff interactions, peer support, prayer) for survivors Survivors were at risk for onset of depression and PTSD symptoms, particularly flashbacks
Blattman et al.^22^	Monrovia (urban)	Qualitative (interviews) Quantitative (survey)	*N* = 999 (surveys) *N* = 66 (interviews) Age (mean) = 25.4 years old Sample: all male	Intervention	Explored crime reduction strategies through a combination of cognitive behavioural therapy and randomised cash payments to criminally engaged men Combination of cash and therapy was the most effective method in decreasing crime and violence for at least a year
Levey et al.^18^	Monrovia (urban)	Qualitative	*N* = 75 Sample: adolescents (ages 13–18 years old)	Population characteristics related to mental health Adolescents	Explored the impact of parental loss and family separation among youth Factors determining psychosocial and emotional health include the timing of the loss, strength of connection with the deceased parent and the relationship with the surviving parent or caregiver
Fabian et al.^23^	Maryland County (urban and rural)	Qualitative (free-lists, semi-structured interviews, chart reviews, and focus group discussions)	*N* = 54 Sample: adults (ages 19–81 years old)	Culturally constructed screening tool	Designed a screening tool for mental suffering Idioms relating frustration, thinking too much and pressure were incorporated into the final screening tool
Lange et al.^12^	Monrovia (urban) Public schools	Qualitative (focus groups)	*N* = 72 Sample: adolescents	Substance use Adolescents	Assessed role of peers in influencing substance use Direct and indirect influences of peers included peer pressure, bullying and taunts, putting drugs into food and drinks to be unknowingly consumed by their peers and witnessing substance use
Petruzzi et al.^13^	Monrovia (urban) Public schools	Qualitative (focus groups)	*N* = 72 Sample: adolescents	Substance use Adolescents	Explored risk factors for substance use among students Risk factors included emotional instability, gender, fear of academic failure, accessibility to substances and poverty Alternative recreational activities and other programs may help in preventing substance use among Liberian youth

IPV, Intimate partner violence; PTSD, post-traumatic stress disorder.

Articles were coded to capture main themes and characteristics. To ensure that data were accurately extracted, 50% (*n* = 11) of the articles were randomly selected and double-coded (KH and KA). Minimal discrepancies between coders arose and were discussed and resolved via consensus. After ensuring consistency between coders, the remaining 50% of articles (*n* = 10) were single-coded (KH or KA).

To describe the quality of the included studies, we modified an existing appraisal tool^10^ that was previously used to assess datasets that include articles consisting of both quantitative and qualitative approaches. Though some articles incorporated both quantitative and qualitative approaches, authors most commonly fully reported on only one type of data (e.g. a focus group was used to provide initial data to guide survey development, but the main results of the article focused on quantitative data). To account for this, all articles were assessed only once in the quality appraisal and were assigned to either the quantitative or qualitative appraisal tool based on the primary outcomes and data reporting. One author (KH) conducted the quality appraisal process. See Tables 2 and 3 for a full description of the assessment parameters and ratings for each article.

**TABLE 2:** Quality assessment for quantitative studies.

First author, year	Selection procedures	Cultural adaptation of the assessment tool	Outcome assessment	Analysis	Global assessment
Lekskes et al.^21^	W	W	W	W	W
Johnson et al.^7^	S	M	S	S	H
Galea et al.^25^	S	W	S	S	H
Rockers et al.^26^	S	W	S	S	H
Harris et al.^11^	S	W	S	S	H
Johnson et al.^27^	S	W	S	S	H
Vinck and Pham^24^	S	M	S	S	H
Ghailian^19^	M	W	M	M	M
Quiterio et al.^15^	S	W	S	S	H
Borba et al.^3^	S	M	S	S	H
Blattman et al.^22^	M	W	S	M	M

*Source*: Adapted from Ronzi et al.^10^

Each item was rated as S = strong, M = moderate and W = weak. The global descriptive assessment is given based on appraising the items to give a range from lower to higher quality: H = high; M = medium; L = low.

**TABLE 3:** Quality assessment for qualitative studies.

First author, year	Quality of reporting		Methodology				Reliable data analysis methods	Global assessment
	Clear aims	Clear context	Adequate sampling methods	Data collection methods described	Data analysis methods described	Adequate amount of data presented		
Dominguez et al.^17^	Y	Y	Y	P	P	Y	Y	M
Levey et al.^20^	Y	Y	Y	Y	Y	P	Y	H
Prust et al.^14^	Y	Y	Y	P	P	P	Y	M
Levey et al.^18^	Y	Y	Y	P	Y	Y	Y	H
Pullen et al.^16^	Y	Y	Y	Y	Y	Y	Y	H
Rabelo et al.^6^	Y	Y	P	Y	P	Y	P	M
Levey et al.^4^	Y	Y	Y	P	Y	Y	Y	H
Fabian et al.^23^	Y	Y	Y	Y	Y	Y	Y	H
Lange et al.^12^	Y	Y	Y	Y	Y	Y	Y	H
Petruzzi et al.^13^	Y	Y	Y	Y	Y	Y	Y	H

*Source*: Adapted from Ronzi et al.^10^

Each item was rated as Y = yes; N = No; P = Partly; NR = Not reported. The global descriptive assessment is given based on appraising the items to give a range from lower to higher quality: H = high; M = medium; L = low.

## Results

Publication years of the primary citations ranged from 2007 to 2018. The majority of the studies were qualitative in nature (*k* = 9, 42.9%), and the remainder used mixed methods (*k* = 6, 28.6%) or quantitative designs (*k* = 6, 28.6%). Many studies were conducted in Monrovia (Montserrado County), the capital city of Liberia (*k* = 10, 47.6%), whilst others were completed in Lofa (*k* = 1, 4.76%), Nimba (*k* = 2, 9.52%) and Maryland counties (*k* = 1, 4.76%). One study noted that respondents came from 15 counties (*k* =1, 4.76%), and other articles did not further specify locations of respondents (*k* = 5, 23.8%). One study (*k* = 1, 4.76%) noted that participants were selected from both urban and rural settings.

Number of participants in the studies ranged from 17 to 4501. Articles highlighted notable characteristics of the study participants, including: individuals from an Ebola treatment unit (*k* = 1, 4.76%), key informants selected by study staff and local organisations (*k* = 3, 14.29%), individuals selected from areas of high crime (*k* = 1, 4.76%),), individuals identified as current and former substance users (*k* =1, 4.76%), former combatants (*k* = 1, 4.76%) and students from local schools (*k* = 5, 23.81%). See Table 1 for study-specific details.

Regarding quality assessment, 11 articles were evaluated using the quantitative assessment tool. Of these, eight (72.7%) were considered high quality, two (18.2%) were designated as medium quality and one (9.1%) was assessed as low quality. For the primarily qualitative articles (*n* = 10), seven articles (70%) were determined to be high quality, and three articles (30%) were categorised as medium quality. No qualitative articles were classified as low quality.

Common topics covered in the articles included adolescent mental health, intervention and assessment and post-conflict impacts. One article focused on mental health impacts after recovery from Ebola. Each of these themes will be discussed further below.

### Adolescent mental health

Emphasis on adolescent mental health comprised a significant bulk of the included literature (*k* = 12; 57.14%). Subcategories that were frequently explored included substance use and vulnerabilities of youth as associated with mental health outcomes. In addition, one article focused on resilience factors amongst Liberian adolescents.

#### Adolescent substance use

Six articles^11,12,13,14,15,16^ (28.57%) focused on adolescent substance use, assessing both rates and associated behaviours stemming from substance use. Harris et al. sampled secondary school students in Monrovia and found that alcohol was the commonly used substance, additionally reporting that marijuana was more frequently used by older respondents (19 years and above) and by males.^10^ Other authors^12,13,14,15^ described risk factors associated with adolescent substance use and identified the following variables: participation in war, forced drug use, peer influence (including bullying and peer pressure), sexual behaviours and sexual violence, emotional instability, gender, fear of academic failure, accessibility to substances, poverty and unintentional drug use. Conversely, a main protective factor against initiation of substance use included school attendance^16^. Consequences of adolescent substance use included individual impacts (e.g. substance dependence, physical health impacts, negative social ramifications, misbehaviour) and community impacts (e.g. crime, violence).^14,16^ Use of substances was identified as a means of coping with anxiety and assisting with social facilitation^15^. Areas of recommended future research include exploration of cultural factors related to substance use^11,16^ and focus on the use of substances in school settings.^16^ Suggested interventions include increased training on treatments for substance use for practitioners,^14^ education for students related to minimising risk from sexual activity and substance use,^15^ access to alternative recreational activities^13^ and focus on the role of peers in adoption of risky behaviours.^12^

#### Vulnerabilities of youth associated with mental health outcomes

Five manuscripts^3,17,18,19,20^ (23.8%) reported vulnerability factors amongst Liberian adolescents, whilst also describing needs inherent in this population. Many of these vulnerabilities stem from exposure to conflict, as well as daily experiences in post-conflict society. These experiences include involvement in violence, substance use, exposure to sexual violence, poverty, infectious disease and parental loss^3,17,18^ Ghailian reported that trauma exposure was negatively associated with adolescent prosocial behaviour.^19^ Compounding these vulnerabilities are risk factors such as negative influence of peers and lack of safety or family support in navigating social contexts.^17^ Past work noted that significant functional impairment stemming from unaddressed mental health needs creates difficulty for youth in accessing or benefitting from protective factors.^3^ To buffer these vulnerabilities, recommendations include: strengthening community-based support, integration of mental healthcare into medical services and community activities,^3^ focusing on specific needs of orphans^18^ and increasing youth access to counseling, education and skills training, in addition to medical care.^20^ Opportunities for research include focus on identification of types of trauma experienced by adolescents, as well as exploration of gender differences in responding to traumatic events.^19^

#### Resilience

One article reported factors that increase resilience amongst youth, observing that school enrollment resulted in greater adaptive functioning and better ability to manage emotion regulation, cognitive flexibility and social intelligence.^4^ No other publications investigating this topic emerged in our search.

### Interventions and assessment

Two articles^21,22^ (9.52%) emphasised psychological interventions previously used in Liberia. Lekskes and colleagues enrolled a sample of women who experienced war-related and sexual violence in Liberia to compare the effectiveness of two psychosocial interventions. They found that trauma counseling was more effective than support and skills training in treating trauma symptoms.^21^ The authors recommended that further research into programs that address both mental health symptoms and socioeconomic stressors is necessary. A separate article piloted an intervention to reduce crime by using a combination of cognitive behavioural therapy (CBT) and cash payments to alter behaviour amongst a sample of 999 criminally engaged men (i.e. individuals engaged in part-time theft and drug dealing).^22^ Results indicated that a combination of cash and therapy was the most effective method in decreasing crime and violence for at least 1 year. Additional exploration into mechanisms of sustaining these positive findings, as well as use of therapies outside of CBT, was suggested.

One article^23^ (4.76%) described efforts to design a screening tool to capture distress amongst Liberians. Using a mixed-methods approach of free-lists, semi-structured interviews, chart reviews and focus group discussions, these authors created an assessment with culturally specific idioms related to frustration, thinking too much and feelings of pressure; continued validation of this tool was identified as next steps.^23^

### Post-conflict impacts

Five articles^7,24,25,26,27^ (23.81%) focused on post-conflict influences related to mental health, with subthemes of trauma and needs of former combatants.

#### Trauma

Three articles^24,25,26^ (14.29%) described various outcomes from trauma: exposure to intimate partner violence, prevalence of post-traumatic stress disorder (PTSD) and impacts of trauma in the context of community. Vinck and Pham studied the association of intimate-partner physical violence and potentially traumatic war-related events amongst Liberian men and women.^24^ The study found a relationship between intimate-partner violence and symptoms of PTSD and depression for women, as well as an association between perpetrating intimate-partner physical violence and symptoms of PTSD and depression for men. Galea et al. examined the geographical distribution of PTSD in Nimba County, Liberia reports that the prevalence of PTSD (48.3%; 95% confidence interval = 45.7, 50.9; *n* = 664) was consistent with the geographic patterns of conflict across the country.^25^ Rockers et al. noted the association between community characteristics and post-traumatic stress symptoms in a resettled post-conflict population in Liberia.^26^ These authors described both risk and protective factors on community levels that have individual-level impact (e.g. living in a village with a higher mean number of traumatic experiences resulted in higher symptom scores on self-report measures). These authors recommended that future works attend to community characteristics and village level interventions that may impact individual well-being.

#### Former combatants

Two articles^7,27^ (9.52%) discussed mental health effects and needs specific to former combatants. Johnson et al. discussed the association between previous head injury and mental health symptoms, finding that former combatants with reported head injuries had an increased risk of major depressive disorder (MDD), suicidal ideation and substance abuse.^27^ Another article similarly described that former combatants tended to have higher rates of MDD, suicidal ideation and PTSD.^7^ They also reported that individuals who experienced sexual violence had an elevated risk beyond those who only experienced combat. Both articles emphasised the importance of focusing on the mental health needs of this group, as well as increased attention to the role of sexual violence amongst combatants.

### Ebola

One article focused on the mental health impacts of Ebola.^6^ Mental health risk factors for survivors included stigma, exposure to death, isolation from family, negative interactions with their medical care providers; conversely, positive provider interactions, peer support and prayer were associated protective factors. The authors found that survivors were at risk for the onset of depressive and trauma symptoms, particularly flashbacks, and suggested the importance of facilitating post-treatment reintegration into communities to promote positive mental health outcomes.

## Discussion

This study presents findings on the current state of the literature regarding mental health and substance use in post-conflict Liberia. Of note, review of four databases resulted in only 21 articles that captured these concepts, suggesting that there is a dearth of literature about the current landscape of mental healthcare in Liberia. This offers ample opportunity for researchers to investigate mental health needs amongst the Liberian population and current practices, as well as a need to strengthen the cadre of researchers who are interested in and prepared to address these questions. After a significant disruption of its education system, Liberia has made gains in increasing access to and opportunities for schooling, which in turn promotes advancement in education and a subsequent pathway for research capability.^28^ Liberia is simultaneously rebuilding its health system, which was weakened by conflict and challenged by the Ebola outbreak in 2014.^29^ One key feature of a well-functioning health system is its ability to track and gather information that guides key decisions, which includes ongoing health research as a main component.^30^ Efforts to train and integrate local staff into these roles are advised.^31^

In our review, one of the most common topics addressed in the existing literature was adolescent mental health, particularly themes associated with vulnerabilities that youth face because of conflict exposure and issues related to youth substance use. Emphasis on adolescent needs is critical as it is estimated that 10% – 20% of children worldwide experience mental health disorders^32^ and juvenile onset of mental health disorders is associated with worsened outcomes in adulthood.^33^ Early intervention and prevention may mitigate personal and societal challenges that stem from early onset of mental health problems.^34,35^

Such focus is critically important in light of the recent events that Liberia has faced. Adolescents impacted by conflict commonly experience distress and are at risk for the development of subsequent symptoms of mental illness and negative impacts on well-being.^36,37,38^ Youth exposed to repeated stressors are more likely to develop disorders such as post-traumatic stress disorder, anxiety disorders and mood disorders.^34^ In addition, conflict exacerbates daily stressors (e.g. poverty) which compounds negative impacts on well-being.^37^ Articles included in this study highlighted the vulnerabilities of Liberian youth,^3,17,18^ many of which have resulted in negative emotional health impacts. Both global^36,38^ and Liberia-specific^3,20^ literature elucidate factors that assist in buffering these impacts, such as strengthening social support from both family and the community, including teachers and peers. Implementing these recommendations, with particular emphasis on the positive role that education serves, may act as a key rebuilding effort.

Substance use behaviours, as well as risk factors for use initiation, were also commonly discussed in the literature. It is known that adolescents exposed to violence and conflict are more likely to engage in substance use,^39,40^ and this same pattern appears to be true in Liberia.^12,13,14,15^ Substance use disorders (SUDs), when comorbid with other mental conditions, may result in worsened physical and mental health.^41,42^ As some Liberian youth may already face heightened risk of psychiatric disorders, it is necessary to continue monitoring prevalence of use, risky substance use, and protective factors. Participation in school, which was identified as a protective factor to promote positive mental health gains, was also observed as one mechanism to buffer some of the risks associated with youth substance use.^16^

Whilst there is significant focus on youth, further assessment and exploration of the mental health needs of adult and elderly populations are also warranted. Past research on the effects of traumatic events, including conflict, on middle- and older-aged adults is mixed. Although it is generally suggested that older adults are more resilient in the face of such stressful experiences,^43,44^ certain factors (e.g. economic impact, past experiences) may result in greater susceptibility to impacts from stress, trauma and conflict.^43,45^ As adults are not exempted from developing psychiatric conditions, greater exploration of the needs of this population remains. Finally, efforts to improve and bolster adult mental health may also positively impact the mental health needs of children and youth, as past literature notes the parallel relationship between child and adolescent distress and maternal mental health.^46,47^

Our review also demonstrates that there are many topics that remain unexplored. For example, whilst three articles focused on adapted interventions and measures that are used in Liberia, there is little evidence of other interventions that may assist in mental health recovery efforts. Adaptation is a key feature in ensuring that interventions are: (1) relevant and efficient in varying cultures and contexts; (2) aligned with existing community understanding of mental health^48,49,50^ and (3) serving as a means to transfer knowledge into practical application for treatment. Similarly, much of the existing data used in the literature come from urban areas, particularly the capital city of Monrovia. Exploration of both similarities and differences related to rates of mental illness, impacts of traumatic events on communities and nuanced understanding of ways that mental health and substance use is described and understood is necessary to ensure that interventions maintain relevance in varying cultural and geographical communities. For example, Rockers et al. described community level factors that affect individual level mental health, whilst suggesting that living in different geographical locations resulted in differing experiences and mental health outcomes.^26^ Finally, many of the included articles used qualitative designs to gather deep knowledge about previously underexplored concepts.^16,18^ One area for continued scholarly exploration includes the use of other research methods to expand, replicate and/or confirm findings in broader samples.

Surprisingly, there is very little literature specifically assessing mental health outcomes associated with exposure to Ebola (i.e. personal, societal and/or medical impacts). The one article included in this review was also limited to individuals released from Ebola Treatment Units, though impacts from Ebola rippled throughout the entire population. Mass disaster and community upheaval are often associated with negative mental health impacts^51,52^ and may require psychosocial intervention to promote functioning. Longitudinal investigations regarding the impact of the Ebola outbreak may give insight on how to best direct limited resources and minimise long-term impacts.

Limitations of this work include its inclusion criteria, namely the requirement that sources for this review exist as empirical articles. Whilst it is possible that other literature relating to mental health exists (e.g. governmental documents, works outside of formal databases), the purpose of this review was to specifically assess the empirical literature focusing on Liberian mental health, in part to gauge scholarly activity focused on these topics. Another consideration is that this work required that included articles be written in English. Whilst English is commonly spoken in Liberia, it is possible that other authors or organisations may have published in other languages. Lastly, the quality appraisal of the included articles was conducted by one reviewer; however, we specifically included only peer-reviewed articles to provide an initial layer of quality assurance.

In summary, our review indicates that there is a small, but growing and quality literature regarding mental health and substance-use care, needs and practices in Liberia. Expansion beyond these domains is needed, both to guide Liberia’s current needs, and to provide practitioners with guidance for practice. Continued investment in psychiatric care and research will be an important component of recovery efforts in Liberia.
